# The p factor: genetic analyses support a general dimension of psychopathology in childhood and adolescence

**DOI:** 10.1111/jcpp.13113

**Published:** 2019-09-20

**Authors:** Andrea G. Allegrini, Rosa Cheesman, Kaili Rimfeld, Saskia Selzam, Jean‐Baptiste Pingault, Thalia C. Eley, Robert Plomin

**Affiliations:** ^1^ Social, Genetic and Developmental Psychiatry Centre Institute of Psychiatry, Psychology and Neuroscience King's College London London UK; ^2^ Division of Psychology and Language Sciences University College London London UK

**Keywords:** Childhood psychopathology, behavioural genetics, genomics

## Abstract

**Background:**

Diverse behaviour problems in childhood correlate phenotypically, suggesting a general dimension of psychopathology that has been called the p factor. The shared genetic architecture between childhood psychopathology traits also supports a genetic p. This study systematically investigates the manifestation of this common dimension across self‐, parent‐ and teacher‐rated measures in childhood and adolescence.

**Methods:**

The sample included 7,026 twin pairs from the Twins Early Development Study (TEDS). First, we employed multivariate twin models to estimate common genetic and environmental influences on p based on diverse measures of behaviour problems rated by children, parents and teachers at ages 7, 9, 12 and 16 (depressive traits, emotional problems, peer problems, autism traits, hyperactivity, antisocial behaviour, conduct problems and psychopathic tendencies). Second, to assess the stability of genetic and environmental influences on p across time, we conducted longitudinal twin modelling of the first phenotypic principal components of childhood psychopathological measures across each of the four ages. Third, we created a genetic p factor in 7,026 unrelated genotyped individuals based on eight polygenic scores for psychiatric disorders to estimate how a general polygenic predisposition to mostly adult psychiatric disorders relates to childhood p.

**Results:**

Behaviour problems were consistently correlated phenotypically and genetically across ages and raters. The p factor is substantially heritable (50%–60%) and manifests consistently across diverse ages and raters. However, residual variation in the common factor models indicates unique contributions as well. Genetic correlations of p components across childhood and adolescence suggest stability over time (49%–78%). A polygenic general psychopathology factor derived from studies of psychiatric disorders consistently predicted a general phenotypic p factor across development (0.3%–0.9%).

**Conclusions:**

Diverse forms of psychopathology generally load on a common p factor, which is highly heritable. There are substantial genetic influences on the stability of p across childhood. Our analyses indicate genetic overlap between general risk for psychiatric disorders in adulthood and p in childhood, even as young as age 7. The p factor has far‐reaching implications for genomic research and, eventually, for diagnosis and treatment of behaviour problems.

## Introduction

The p factor, analogous to the concept of general intelligence (‘g’), reflects the observation that individuals who score highly on certain psychopathological traits also score highly on others (Caspi et al., 2014). Recent research suggests that this single continuous dimension can, in part, summarise and explain liability to a wide range of psychopathologies in childhood.

Interest in the p factor stemmed initially from high levels of psychopathological comorbidity in adults. The co‐occurrence of psychiatric disorders is strikingly high, with up to 50% of individuals diagnosed with a mental illness going on to develop two or more comorbidities in a 12‐month period (Kessler et al., 2005). Already during childhood and adolescence, forms of psychopathology are often comorbid. A recent report found that 1 in 20 British young people under 20 years of age met criteria for 2 or more mental disorders (NHS Digital 2017).

Quantitative genetic research suggests that shared genetic factors contribute substantially to the observed co‐occurrence of psychopathological traits (Plomin, DeFries, Knopik, & Neiderhiser, 2016). Several multivariate twin and family studies have replicated the finding that a common genetic factor influences a wide range of emotional and behavioural problems in childhood (Lahey, Van Hulle, Singh, Waldman, & Rathouz, 2011; Pettersson, Larsson, & Lichtenstein, 2016; Tackett et al., 2013; Waldman, Poore, van Hulle, Rathouz, & Lahey, 2016). Many studies have investigated developmental genetic effects on specific psychopathological traits in childhood (e.g. Pingault et al., 2015), yet little is known about the genetic and environmental architecture of general psychopathology across development. Stability and change in p across time and the extent to which genetic influences drive age‐related patterns remain largely unknown. Here, for the first time, we systematically investigate p across diverse ages, raters and measures in childhood and adolescence.

It is also unknown to what extent a general p factor across earlier development relates to adult psychopathology. In addition to genetic analyses using the twin and family designs, polygenic scores are a new genomic tool that can be used to test for shared genetic effects across traits. Polygenic scores are constructed by aggregating genetic risk across thousands of genetic variants, thus indexing the genetic liability that each individual carries for a specific trait. A landmark study in the field of psychiatric genetics (International Schizophrenia Consortium et al. 2009) first showed that a polygenic score for schizophrenia was also associated with bipolar disorder, suggesting a shared genetic component underlying these two disorders, which has been substantiated further more recently (Cross‐Disorder Group of the Psychiatric Genomics Consortium et al. 2019). Several studies have used polygenic scores for schizophrenia, ADHD and other psychiatric disorders to predict general psychopathology in childhood. An increasing amount of evidence converges on the finding that few polygenic effects specific to individual aspects of psychopathology remains after conditioning on the p factor (Brikell et al., 2017; Jones et al., 2016, 2018; Riglin et al., 2018). These studies also suggest that genetic risk for psychiatric disorders emerges in childhood, in the form of continuously measured behaviour problems. More recently, a study using different genomic methods provided evidence for a ‘polygenic p’ factor (Selzam, Coleman, Caspi, Moffitt, & Plomin, 2018). However, no studies to date have empirically related ‘polygenic p’ to ‘phenotypic p’ or systematically tested the architecture of p across development and across different raters.

Here, we investigated the structure of general psychopathology across childhood and adolescence. Our study has three aims:

1. Investigate the genetic architecture of p in childhood through common pathway twin models across ages and raters.
2. Test the stability of p across childhood and adolescence through longitudinal quantitative genetic analysis of first principal components of psychopathology across ages (7, 9, 12 and 16) and raters (parent, teacher and self‐ratings).
3. Estimate associations between childhood phenotypic p and adult polygenic p. The latter can be constructed by principal component analysis of polygenic scores for mostly adult psychiatric disorders created for each TEDS participant.

## Methods

### Sample

The sampling frame is the Twins Early Development Study (TEDS), a multivariate, longitudinal study of >10,000 twin pairs representative of England and Wales, recruited from 1994 to 1996 births (Haworth, Davis, & Plomin, 2013). The following exclusions were applied: extreme perinatal conditions, severe medical conditions, uncertain zygosity and unknown gender. Analyses were conducted on a subsample of unrelated individuals with available genotype data and their cotwins (*N* = 7,026). Genomic analyses were limited to unrelated individuals (one twin from each pair).

### Genotyping

Data were available for 3,057 individuals genotyped on the Affymetrix GeneChip 6.0 and 3,969 individuals genotyped on HumanOmniExpressExome‐8v1.2 arrays. Typical quality control procedures were followed (e.g. samples were removed based on call rate <0.98, MAF < 0.5%). Genotypes from the two platforms were separately imputed and then harmonised (for detail see Selzam et al., 2018).

### Measures

Twins Early Development Study (TEDS) measures have been described previously (Haworth et al., 2013). Measures administered at ages 7, 9, 12 and 16 were included in our analyses. Some of these measures (e.g. peer problems, prosocial behaviour (reversed), autism traits) have not previously been used in other studies of general psychopathology, but we adopted a hypothesis‐free approach in an attempt to capture a general trait that is pervasive across diverse domains. For similar reasons, we included all measures available at each age, even though some measures (e.g. aggression) were available only at one age. Table 1 summarises the measures included in this study. Due to the wide range of ages, raters and measures used in the analyses; for information on response rates, please see Haworth et al. (2013).

**Table 1:** Summary of psychopathology measures available in the Twins Early Development Study (TEDS)

Construct	Measure	Age/reporter	Reference
ADHD behaviours	Conners Parent Rating Scale	P12, P16	Conners (2003)
Aggression	Reactive‐Proactive Aggression Questionnaire	P9, T9	Dodge and Coie (1987)
Anxiety‐related Behaviours	ARBQ	P16	Eley et al. (2003)
Autism traits	Autism traits	P7, T7	DSM‐IV criteria items
	Childhood Autism Spectrum Test (CAST)	C9, P9, T9, C12, P12, T12	Scott et al. (2002) and Williams et al. (2005)
	Autism Quotient (AQ)	C16, T16	Baron‐Cohen et al. (2001)
Callous Unemotional Traits	Callous Unemotional Scale	P16	Kimonis et al. (2008)
Conduct Problems	Strengths and Difficulties Questionnaire (SDQ)	P7, T7, C9, P9, T9, C12, P12, T12, C16, T16	Goodman (1997)
Depressive Symptoms	Moods and Feelings Questionnaire (MFQ)	P12, C16, T16	Angold et al. (1995)
Emotional Problems	Strengths and Difficulties Questionnaire (SDQ)	P7, T7, C9, P9, T9, C12, P12, T12, C16, T16	Goodman (1997)
Hyperactivity	Strengths and Difficulties Questionnaire (SDQ)	P7, T7, C9, P9, T9, C12, P12, T12, C16, T16	Goodman (1997)
Peer Problems	Strengths and Difficulties Questionnaire (SDQ)	P7, T7, C9, P9, T9, C12, P12, T12, C16, T16	Goodman (1997)
Prosocial Behaviour	Strengths and Difficulties Questionnaire (SDQ)	P7, T7, C9, P9, T9, C12, P12, T12, C16, T16	Goodman (1997)
Psychopathic Tendencies	Antisocial Process Screening Device (APSD)	P7, T7, P9, T9, C12, P12, T12	Frick and Hare (2001)

Letters refer to the reporter (C = child self‐report, P = parent report, T = teacher report), and numbers refer to the ages at which measures were available.

For all phenotypes, *z*‐standardised residuals were derived for each scale regressed on sex and age. Standardised scores for each scale were calculated as mean scores, with the requirement of complete data for more than half of the items (i.e. 3 of 4 or 2 of 3). All procedures were executed using RStudio (version 1.1.419; Rstudio 2019).

#### Age 7 measures

We used both parent and teacher ratings of all subscales of the Strengths and Difficulties Questionnaire (SDQ; Hyperactivity, Conduct Problems, Peer Problems, Emotional Problems and Prosocial (reversed; Goodman, 1997), as well as the Antisocial Process Screening Device (APSD; Frick & Hare, 2001) and a measure of autism traits.

#### Age 9 measures

The five subscales of the SDQ and the Childhood Autism Spectrum Test (CAST; Scott, Baron‐Cohen, Bolton, & Brayne, 2002; Williams et al., 2005) were included in the set of self‐, parent‐ and teacher‐reported measures. In addition, we used parent‐ and teacher‐rated APSD and aggression (a mean of proactive and reactive scales) measures (Dodge & Coie, 1987).

#### Age 12 measures

The five subscales of the SDQ, the APSD and the CAST were included in the set of self‐, parent‐ and teacher‐reported measures. Parent reports of the Moods and Feelings Questionnaire (MFQ) assessing depressive traits (Angold, Costello, Messer, & Pickles, 1995) and the Conners’ ADHD behaviour measure (Conners, 2003) were also available.

#### Age 16 measures

The five subscales of the SDQ, MFQ and The Autism Quotient (Baron‐Cohen, Wheelwright, Skinner, Martin, & Clubley, 2001) were available from self‐, parent and teacher reports. Parent‐rated data on Conners ADHD measure, the inventory for the Callous Unemotional scale (Kimonis et al., 2008) and the Anxiety‐related Behaviors Questionnaire (Eley et al., 2003) were also included.

### Statistical analyses

#### Common pathway twin models of behaviour problem measures for each rater at each age

To estimate the genetic and environmental influence on phenotypic variance in general psychopathology and to examine loadings of individual psychopathology measures on p, we conducted multivariate twin model‐fitting analyses. In the twin design, differences in within‐pair trait correlations for monozygotic (MZ) and dizygotic (DZ) twins are used to estimate genetic, shared environmental and nonshared environmental effects on traits. Greater MZ than DZ similarity indicates additive genetic influence (A). Within‐pair similarity that is not due to genetic factors is attributed to shared environmental influences (C). Nonshared environment (E) accounts for individual‐specific factors that influence differences among siblings from the same family, plus measurement error. We considered genetic and environmental associations between all psychopathology measures at each age and separately for each rater. Specifically, we fit the data to the common pathway model (Rijsdijk, 2014). This is a multivariate twin model, in which common genetic and environmental variation influence all measures via a single common latent (p) factor. The model allows the estimation of genetic and environmental influences on a common factor (p) and of the factor loadings of each measure of psychopathology on the latent liability (p). The common pathway model also allows the estimation of genetic and environmental variance in each trait that is independent of the common factor.

#### Longitudinal twin analysis: Cholesky decomposition of phenotypic principal components

We performed a Cholesky decomposition of the parent‐rated phenotypic p principal components, allowing for the investigation of stability and innovation in the genetic and environmental influences on our measures of p across the four ages. We focused on parent‐rated data since measures were much more consistent across time than for self‐report and teacher report. The first genetic factor (A1) represents genetic influences on p at age 7. The extent to which these same genes also influence p at ages 9, 12 and 16 is also estimated, and is represented by the diagonal pathways from A1 to the other variables. The second genetic factor (A2) represents genetic influences on p at age 9 that are independent of those influencing age 7. The extent to which these genes also influence p at ages 12 and 16 is also estimated. The third genetic factor (A3) indexes genetic influences on p at age 12 that are independent of genetic influences shared with the previous ages. The impact of these genes on age 16 general psychopathology is also estimated. Finally, the fourth genetic factor (A4) represents residual genetic influences on age 16 general psychopathology. The same decomposition is done for the shared environmental and nonshared environmental influences (C1–4 and E1–4, respectively). All twin model fitting analyses using full‐information maximum likelihood were carried out with structural equation modelling software OpenMx (Neale et al., 2016).

#### Extracting p: Principal Component Analyses (PCA)

In preparation for longitudinal analyses and genomic prediction analyses, we obtained the first principal component (1st PC) of behaviour problem phenotypes at each age separately for child, parent and teacher ratings. Only individuals with complete data were used to generate PCs, as PCA does not allow for missing data. We report full results from PCA, which in themselves give insights into the phenotypic architecture of p in childhood. The variance explained by the first PC suggests how much the p factor underpins diverse forms of psychopathology, and loadings of each measure on the first PC indicate the extent to which variables reflect general psychopathology.

We also obtained the first PC from polygenic scores for psychiatric disorders (polygenic p). We used publicly available genome‐wide association summary statistics for eight major psychiatric traits: autism spectrum disorder (Grove et al., 2019), major depressive disorder (MDD; Wray et al., 2018), bipolar disorder (BIP), schizophrenia (SCZ; Pardiñas et al., 2018), attention deficit hyperactivity disorder (ADHD; Demontis et al., 2019), obsessive–compulsive disorder (OCD; International Obsessive Compulsive Disorder Foundation Genetics Collaborative (IOCDF‐GC) and OCD Collaborative Genetics Association Studies (OCGAS), 2018), anorexia nervosa (AN; Duncan et al., 2017) and posttraumatic stress disorder (PTSD; Duncan et al., 2018). For each psychiatric disorder, polygenic scores for each TEDS participant were created in LDpred (Vilhjálmsson et al., 2015), assuming a fraction of causal markers of 1 (analysis steps were similar to Selzam et al., 2018).

#### Assessing the association between the polygenic 1st PC and the phenotypic 1st PC across childhood and adolescence

To assess the extent to which the genetic predisposition for a general psychopathology factor relates to p in childhood, we performed ordinary least square regression analyses of phenotypic p on polygenic p at each age separately by each rater. Age, sex and the first 10 genomic principal components were regressed from all dependent and independent variables, and standardised residuals were used in all linear models.

## Results

### Common pathway twin models

**Figure 1:**
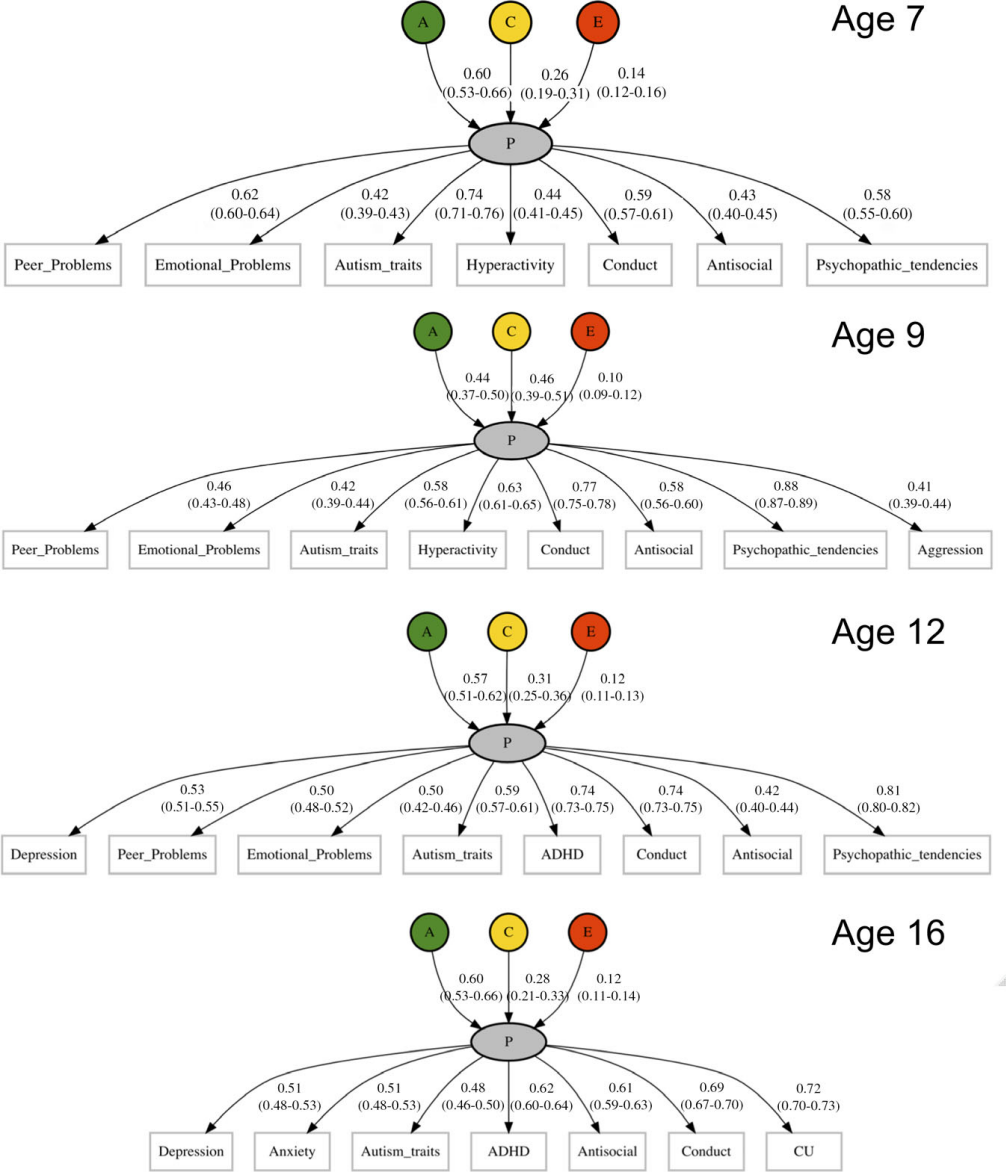
Common pathway twin models of p (parent rated) at ages 7, 9, 12 and 16

Common pathway twin models showed substantial heritability for the p factor at each age for all raters (50%–60%). See Figure 1 for parent‐rated measures and Figure S1 for teacher‐rated and child‐rated measures. Figure S2 summarises the heritability estimates for the common factor at each age and for each rater. Shared environmental effects were moderate for the parent‐rated common factors (~30%; Figure S1), absent for the teacher‐rated common factors (~0%; Figure S1) and weak for the self‐rated common factors (~15%, declining with age; Figure S1). Autism traits, conduct problems, antisocial behaviour and psychopathic tendencies loaded the highest on the parent‐rated and teacher‐rated common factors, while emotional problems, depression and anxiety loaded the highest for the child‐rated p factor. We also found substantial specific genetic and environmental variance for all measures suggesting unique influences on psychopathological measures beyond the p factor. See Table S1 for full estimates of common and specific genetic and environmental influences. Table S1 also contains full model‐fitting results, and sample sizes of measures, which ranged from 2,216 to 5,592 twin pairs who also had genotype data. See Table S2 for model fit statistics.

### Cholesky decomposition of p across development

**Figure 2:**
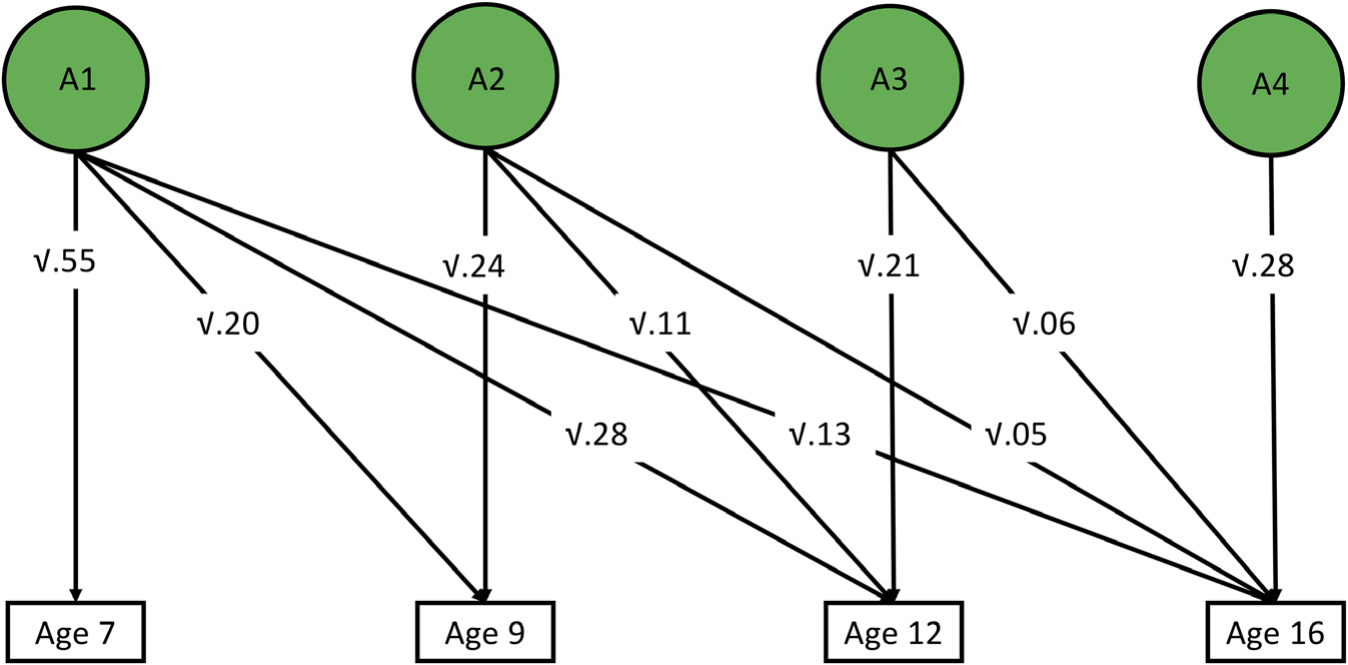
Additive genetic influences on parent‐rated p across age, derived from longitudinal twin model‐fitting (Cholesky decomposition)

The Cholesky decomposition of principal components suggests stability of genetic effects on general psychopathology across childhood and adolescence, in addition to new genetic components at each age, as shown in Figure 2 for parent ratings. Figure S5 shows genetic correlations derived from a correlated factor solution. Age‐to‐age genetic correlations derived from these results are high, ranging from 0.49 to 0.78 (see Figure S5). Figures S3 and S4 present the Cholesky model‐fitting results for shared and nonshared environmental variance components, respectively. Figures S6–S15 indicate phenotypic correlations among psychopathology measures at all ages and for all raters. These correlations are notably similar to genetic correlations from the Cholesky model. Figure S16 shows phenotypic correlations between principal components across age and raters. Parent‐rated correlations were the strongest, ranging from .47 to .68, while child and teacher‐rated correlations were somewhat weaker, but still substantial (i.e. ~.3 to ~.4 for both). Cross‐rater correlations were strongest for child‐ and parent‐rated p factors, ranging between ~.3 and ~.4, and weakest between teacher‐rated and child‐rated p factors (~.1 to ~.2). Table S3 lists loadings of observed measures on first principal components, which shows that loadings are consistently substantial for all measures, ages and raters. The first unrotated principal component of phenotypic measures accounted for 40% to 50% of the variance across ages and raters (see Table S4, which also shows the sample sizes for each 1st PC, which ranged from 1,391 to 4,490).

### Prediction of phenotypic p with polygenic p

**Figure 3:**
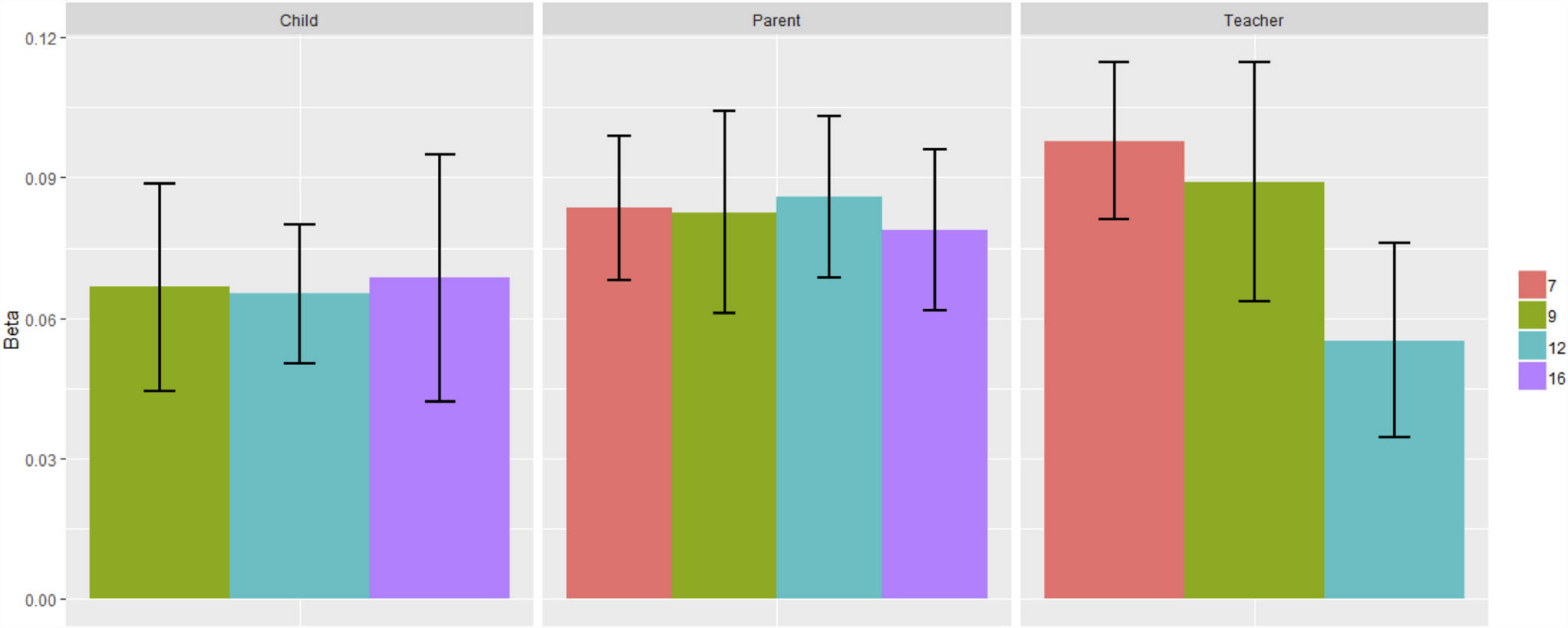
Prediction of phenotypic p with polygenic p by ages and raters. *Note*: Error bars represent ± 1 standard error

A polygenic p score defined as the first unrotated principal component of polygenic scores for mostly adult psychiatric disorders was significantly associated with phenotypic p scores in childhood, predicting 0.3%–0.9% of the variance across ages and raters. See Table S5 for full polygenic prediction results. Prediction was generally consistent across ages and raters, although standard errors are largely overlapping (see Figure 3). Figure S17 shows correlations between the polygenic scores in TEDS used to derive polygenic p. Although these correlations are modest (0.01–0.32), the first principal component of polygenic scores from psychiatric traits explained up to 20% of the polygenic score variability. The loadings on polygenic p (shown in Figure S18) were all above 0.3, apart from obsessive–compulsive disorder (0.13) and posttraumatic stress disorder (0.18). This could be because the GWA summary statistics for these disorders were derived from smaller samples than the others. Analyses applying to differentially powered summary statistics for the same traits to TEDS data have demonstrated that, as GWA study sample sizes increase, factor loadings on a polygenic p factor are likely to approach those derived from family studies (Selzam et al., 2018).

## Discussion

For the first time, we systematically quantified the extent to which a single common factor relates to diverse forms of psychopathology across childhood and adolescence using phenotypic, genetic and genomic methods. Phenotypically, our results parallel previous findings, suggesting a common psychopathology factor. We show that p emerges consistently across different measures at different ages and raters. Our genetic results support three main conclusions. First, multivariate twin analyses revealed that 48%–80% of the variance in the common factor was due to genetic influences, depending on age and raters considered. It is important to note, however, that although we found a consistent and stable genetic p factor across childhood and adolescence, substantial unique genetic and environmental influences indicate that there are also genetic components specific to each trait and each age beyond p. Second, longitudinal twin model fitting showed that this genetic p factor was stable across time. Third, polygenic prediction analyses demonstrate that there are shared genetic influences connecting childhood psychopathology to general risk for (mostly) adult psychiatric disorders. Even though variance predicted is low (i.e. ~1%), effect sizes are within the expected range considering previous research in this area (e.g. Riglin et al., 2018; Grotzinger et al., 2019; see below). In sum, these analyses provide further evidence that a common genetic substrate permeates the landscape of psychopathology, across measures, ages and raters.

Our common pathway twin modelling analyses, for which we adopted a hypothesis‐free approach to the inclusion of measures, show that diverse psychopathological traits contribute to p. Furthermore, it is commonly acknowledged that all psychopathological traits are dimensional traits both at the phenotypic and genetic levels (Plomin, Haworth, & Davis, 2009). Future research might investigate the extent to which p extends to other behavioural domains. For example, suggestive evidence of links between p and personality has begun to emerge (Rosenström et al., 2018). In addition, instead of testing competing factor structures, we focused on the common pathway model, since the present study aimed to investigate the most parsimonious highest order part of the hierarchy that we call p. This is further justified by evidence for correlations and heterotypic sequential comorbidity across the internalising and externalising domains (Caspi & Moffitt, 2018).

Differences between raters in our common pathway twin analyses suggested some additional insights. First, inspection of the loadings of psychopathology measures revealed that ‘externalising’ problems relating to conduct and antisocial behaviour contributed most to parent‐ and teacher‐rated common factors, whereas ‘internalising’ problems such as depression and anxiety loaded highest for the child‐rated p factor. This could suggest that parents report on overt behaviours, which might stem from worry and sadness from the child's perspective. Second, we observed that shared environmental influences were moderate for the parent‐report‐based p factor, but negligible for self‐ and teacher‐rated p, respectively. This pattern of results is most likely due to rater bias in that parent ratings are based on a single informant rating both twins, whereas for teacher and self‐ratings different informants rate each twin (Bartels et al., 2004).

Our longitudinal twin model fitting and polygenic scoring revealed substantial genetic influences on stability of general psychopathology across childhood. Our polygenic score results suggest that these stable genetic influences overlap with those underlying adult psychiatric disorders.

In terms of predictive value, effect sizes of our polygenic p score in association with phenotypic p are weak (~1%). However, these are within the expected range for polygenic prediction of psychiatric traits, and consistent with previous literature on polygenic risk and general psychopathology, whereby current polygenic scores for adult psychiatric traits often explain < 1% of the variance in general psychopathology (Riglin et al., 2018), similar to a polygenic score created from a p factor GWAS (Grotzinger et al., 2019). The predictive accuracy of a polygenic p score will increase as the power of single GWAS of psychiatric traits grows, especially when GWAS go beyond DNA arrays consisting of common SNPs to include all DNA variants as assessed by whole‐genome sequencing. In addition, there is increasing evidence that joint multivariate analyses of traits are likely to increase the predictive power of polygenic scores (e.g. Grotzinger et al., 2019; Maier et al., 2018).

Future research could assess influences on different temporal trajectories of p across childhood and adolescence. One study recently showed that polygenic scores for neurodevelopmental disorders (schizophrenia, ADHD) and depression were associated with early adolescent onset depression, whereas later onset depression was only predicted significantly by depression polygenic scores (Rice et al., 2018). This could be repeated with more powerful polygenic p scores.

Notably, some interesting results also emerge about the environment. There are some known general ‘environmental’ risks for psychopathology such as birthweight, birth complications and childhood maltreatment that are associated with diverse neurodevelopmental outcomes (Caspi & Moffitt, 2018; Lim et al., 2018). However, we find that nonshared environmental effects contribute less than genetic effects to the general psychopathology factor and its temporal stability. As has been demonstrated in previous studies of specific psychopathology, nonshared environment is largely time‐specific, and genetic effects clearly contribute more to stability.

Naturally, through the course of multivariate longitudinal studies like TEDS, there are changes in available measures and informants, which in turn can introduce variability in the pattern of results. That is, our measures of p are not perfect indices of general liability to psychopathology, but reflect the specific measures and raters available at each age. This is problematic when estimating genetic and environmental influences on stability and change in p across time. Specifically, any innovation cannot solely be attributed to p, as it will reflect new influences on new measures that were not available at the previous age. This criticism is difficult to overcome even with the availability of consistent data: exactly the same measure at different time points does not necessarily reflect the same thing. We consider that the availability of varied measures is a strength rather than a limitation of the present study because this means that our strong evidence for genetic p and genetic stability for p emerges despite the use of different measures. In the cognitive literature on g, this phenomenon is known as the *indifference of the indicator* – any set of diverse cognitive measures yields a strong g factor (Spearman, 1904). Factor loadings were consistently substantial, not only across measures but also across ages and raters. Importantly, the phenotypic correlations between first principal components across time (ranging between ~0.5 and ~0.7) suggest that p indexes a consistent core psychopathology trait.

The fact that we can predict childhood p using polygenic p derived from typically adult case–control genome‐wide association studies has several interesting implications. First, it suggests that in young children there are already continuous manifestations of genetic risk for adult case–control psychiatric disorders that are unmeasured in our population‐based, developmental sample. Therefore, this extends the insight from twin analyses within our sample that genetic risk for psychopathology at age 7 correlates about 0.50 with genetic risk for psychopathology at age 16. In other words, early onset behavioural and emotional problems are early signs of psychiatric genetic risk. This supports other evidence for the usefulness of early intervention for psychiatric problems. The second implication of the genetic overlap between p in childhood and adulthood relates to research design. Specifically, researchers could increase the power of genome‐wide association studies to detect DNA variation associated with general risk for psychopathology by aggregating diverse traits across wide age ranges. One way to implement this is a common factor genome‐wide association analysis using Genomic SEM (Grotzinger et al., 2019). Similarly, the modest power of psychiatric polygenic scores to predict traits in childhood could be enhanced using multitrait frameworks to generate predictors that leverage the shared genetic risk between traits (e.g. SMTpred; Maier et al., 2018).

The current clinical zeitgeist focuses on specificity. The recognition that a common factor transcends diverse aspects of psychopathology in childhood is of primary importance, as this knowledge can inform early detection of children at risk in the general population.

**Key points**

- We investigated the underlying structure of p across diverse measures, ages and raters, and consistently found a substantial genetic component, in line with previous theory.
- We showed that this genetic component is stable across time, with influences in childhood being pervasive across development through to adolescence.
- Genomic analyses revealed shared genetic risk between p in children as young as 7 and general risk for adult psychiatric disorders.
- We provide further evidence that, in addition to residual variation specific to each trait, a common genetic substrate permeates the landscape of psychopathology.

## Supporting information


**Figure S1.** Common pathway twin models for child‐rated and teacher‐rated psychopathology measures by age.
**Figure S2.** Comparison of twin heritability estimates from common pathway models.
**Figures S3–S4.** Shared environmental and non‐shared environmental influences on p (parent‐rated) across age, derived from longitudinal twin model‐fitting (Cholesky decomposition).
**Figure S5.** Correlated factor solution of the longitudinal Cholesky decomposition.
**Figures S6–S15.** Phenotypic correlations among psychopathology measures used to construct phenotypic p factors.
**Figure S16.** Correlations of 1st PCs across ages.
**Figure S17.** Correlations between polygenic scores for psychiatric traits used to construct polygenic p.
**Figure S18.** PCA results for polygenic p‐factor.
**Table S1.** Additional parameters derived from common pathway twin models of childhood psychopathology in TEDS.
**Table S2.** Model fit statistics for common pathway twin models of childhood psychopathology in TEDS.
**Table S3.** Loadings on first principal components of psychopathology measures for each age and rater.
**Table S4.** Variance explained by 1st PCs for each age and rater.
**Table S5.** Association statistics for polygenic p across phenotypic p measures.
